# Management of Posterior Capsular Rent in Cataract Surgery Patients at a Tertiary Hospital in India: A Retrospective Study

**DOI:** 10.7759/cureus.77354

**Published:** 2025-01-12

**Authors:** Ajit K Joshi, Subhashree Panda, Pravin Hankare, Sakshi Patil

**Affiliations:** 1 Ophthalmology, Bharati Vidyapeeth (Deemed to Be University) Medical College and Hospital, Sangli, Sangli, IND

**Keywords:** capsulorrhexis, phacoemulsification, pseudoexfoliation, visual acuity, vitrectomy

## Abstract

Background: Cataracts are the primary cause of preventable blindness worldwide, especially in India. Various surgical procedures are available for treating cataracts. Nevertheless, extreme vigilance is required to avoid associated complications of surgical procedures such as posterior capsular rent (PCR), endophthalmitis, and nucleus drop.

Aim: To identify preoperative risk factors, intraoperative management, and postoperative outcomes in patients with PCR.

Methods: We conducted a retrospective observational study of patients operated on for cataracts by small-incision cataract surgery (SICS) or phacoemulsification from January 2018 to October 2023 at Bharati Vidyapeeth (Deemed to be University) Medical College and Hospital, Sangli, India. Records were collected, and 29 eyes of 29 patients who had had PCR during cataract surgery were evaluated for one month postoperatively. Wilcoxon signed-rank test was used for data analysis.

Results: The average age of patients with PCR was 67.8 years, and 55.17% were men. Sixty-nine percent of cases were of SICS, 31.03% of phacoemulsification, and PCR occurred in 51.7% of cases by an inexperienced surgeon. With PCR, risk factors such as corneal opacity (6.9%), pterygium (13.8%), shallow anterior chamber (3.45%), pseudo-exfoliation (13.8%), and small pupil (3.45%) were found to be linked. Management of PCR was performed in 29 cases. Anterior vitrectomy with intraocular lens (IOL) placement in the bag was done in 20.7% of cases (six cases), while anterior vitrectomy with IOL placement in the sulcus and pars plana vitrectomy (PPV) with IOL placement in the sulcus were performed in 3.45% of cases each (one case each). Anterior vitrectomy with iris-claw lens implantation was performed in 13.8% of cases (four cases). Additionally, 6.9% of cases (two cases) were left aphakic, which were later managed with PPV and scleral-fixated IOL (SFIOL) in one case and iris-claw lens implantation in the other. On post-op day 30, 20 patients (69%) achieved the best corrected visual acuity of 6/9-6/6, eight patients (27.6%) achieved 6/18-6/9, and one patient (3.45%) achieved 6/24. On comparison of preoperative vision with vision on day 30, the Z value was -4.418, and the P-value was less than 0.001, showing a statistically significant difference.

Conclusions: Our study showed that with proper intraoperative management and follow-up, PCR can be managed with excellent visual recovery (96.5% of our cases regained vision 6/9 and better).

## Introduction

Cataracts are one of the major causes of preventable blindness in India and globally, with age-related cataracts being the most common type [1]. The complex interplay of sociodemographic, environmental, and genetic factors plays a role in cataractogenesis. In India, cataracts are the leading cause of blindness, accounting for 62.6% of cases [2]. Although there have been advances in cataract surgery, complications still occur. The most common intraoperative complication is posterior capsular rent (PCR), which refers to any breach in the continuity of the posterior capsule [3,4]. The incidence of PCR ranges from 0.45% to 7.5% [5,6]. The consequences of this complication vary and include difficulty in the placement of the intraocular lens (IOL) in the bag (preferred because it is the most physiological); loss of vitreous (which results in an increased risk of sight-threatening complications including cystoid macular edema, retinal detachment, endophthalmitis, and suprachoroidal hemorrhage); vitreous prolapse (endothelial damage and glaucoma); and vitreous wick syndrome. If patients with PCR are not properly managed, whether they have a vitreous loss or not, they may experience poor visual outcomes and require additional interventions [6,7]. However, prior prediction of its occurrence in at-risk patients depends on factors such as patients having inadvertent head movement; deep set sockets; prominent brows; or exaggerated Bell’s phenomenon (while operating under topical anesthesia); poor visibility during surgery (due to corneal opacity, pterygium, thick arcus senilis, or band keratopathy); reduced workspace (secondary to small pupil, intraoperative miosis, or shallow anterior chamber); excessive anterior chamber depth (due to high myopia or post pars plana vitrectomy); posterior polar cataract; pseudoexfoliation; dense asteroid hyalosis; small rhexis with rhexis block, intraoperative surge (during phacoemulsification); and inexperienced surgeon [3]. Timely diagnosis and proper management with facilities available can reduce morbidity significantly.

At Bharati Vidyapeeth (Deemed to Be University) Medical College and Hospital, Sangli, we provide mandatory surgical training to our residents, which, while essential, contributes to intraoperative PCR complications. Against this backdrop, we aimed to study intraoperative PCR cases occurring at our institute. The study’s objectives included identifying risk factors for PCR, evaluating its management (whether addressed during the same procedure or in a subsequent one), and assessing postoperative visual recovery in patients who underwent small-incision cataract surgery (SICS) or phacoemulsification with intraoperative PCR.

## Materials and methods

This retrospective observational study was conducted by collecting the records of operated cataract patients who had intraoperative PCRs at Bharati Vidyapeeth (Deemed to Be University) Medical College and Hospital, Sangli, India from January 2018 to October 2023. The collection of data was performed after approval from the research committee and the ethical review board of the institute: IEC NO. 546, dated 25 January 2024. This was a record-based study carried out with the aim of evaluating various risk factors, including patient-related, surgeon-related, machine-related, and intraoperative factors. The stage of PCR and the management method were recorded for the same reasons, as was the final visual outcome. The study included all senile cataract patients over 50 who underwent SICS or phacoemulsification and had PCR. Patients with incomplete records were excluded. Written and informed consent for participation (in case of PCR) was taken prior to cataract surgery in all patients.

Twenty-nine eyes of 29 patients who fulfilled the inclusion criteria were included in the study. The study involved the collection of patient-related factors that included demographic data such as age and sex and their probable association with inadvertent head movements during surgery. Systemic and ocular comorbidities were noted. A history of ocular surgery of the other eye, as well as the same eye, was noted. Preoperative vision, including both uncorrected visual acuity (UCVA) and best corrected visual acuity (BCVA), were noted. Anterior segment evaluation records were assessed for the presence of factors such as corneal opacity, pterygium, thick arcus senilis, band keratopathy, small pupil, shallow anterior chamber, and pseudoexfoliation. Records of dilated evaluation, including media clarity, were noted. The grade of cataract and type of cataract were documented, with special emphasis on the presence of posterior polar cataracts. Fundus evaluation was also noted.

Evaluations of surgeon-related factors, including the surgeon’s experience with performing SICS or phacoemulsification, were also noted. Phaco machine-related factors predisposing to PCR, like machine malfunction, were looked for. Microscope functioning throughout the study time was also ascertained. Intraoperative details of patients with PCR were evaluated. This includes the stage of surgery at which the rent occurred. Rents were managed on the table. The vitrectomy, if anterior was sufficient, and the placement of the IOL were reported. Whether the IOL was placed in the bag or sulcus iris or whether scleral fixating was performed was also noted, as was the need for a second intervention. Patients with second intervention records were followed up till 30 days after the second procedure. The vision of each patient was recorded on day 1, day 7, and day 30. Statistical analysis of visual outcomes on day 30 compared to the preoperative visual acuity was conducted using the Wilcoxon signed-rank test with the help of a statistician.

The record of the postoperative evaluation on day 30 was searched for any signs of complications related to PCR. Non-contact tonometry records for elevated intraocular pressure were also looked for, as were anterior segment evaluation records for uveitis and corneal edema. A detailed fundus evaluation was noted for retinal detachment and cystoid macular edema. Macular optical coherence tomography was also performed on day 30, in suspected cases of pseudophakic cystoid macular edema.

## Results

A total of 29 patients were found to have PCR intraoperatively in our hospital during the study period. This group comprised eight patients aged between 50 and 59 years, nine patients aged between 60 and 69 years, eight patients aged between 70 and 79 years, and four patients aged between 80 and 89 years. Thus, the majority of individuals fell within the age range of 60 to 69 years. The average age was 67.76 years. Among the total, there were 13 females and 16 males.

Fifteen patients did not have any ocular comorbidities, and 16 patients did not have any systemic comorbidities. One patient had lens-induced glaucoma preoperatively. One had a small pupil. Seven patients had previously undergone surgery on the other eye. None of the patients had a history of any ocular surgery on the same eye. Various systemic comorbidities found were as follows: hypertension (seven patients), ischemic heart disease (five patients), and type 2 diabetes mellitus (T2DM) (four patients). One patient had a sulpha drug allergy. Among the SICS patients, PCR was more common (20 cases) than phacoemulsification (nine cases).

Inexperienced surgeons performed in 15 cases, whereas PCR occurred at the hands of experienced surgeons in 14 cases. Preoperatively, we found the anterior segment to be normal in most cases (18). In the other cases, important findings included different kinds of opacities (one nebular and one streak), pterygium (four cases), pseudoexfoliation (four cases), and a shallow anterior chamber (one case). PCR was most commonly seen in posterior subcapsular cataracts (19 patients), followed by mature cataracts (five patients) and by immature (three patients) and hypermature cataracts (one patient), and pseudo posterior polar cataracts (one patient), respectively. The phaco machine was functional, and the microscope functioned properly on all occasions. Preoperatively, fundal details could not be visualized because of cataracts in 10 cases with normal B scans. These patients underwent surgery, but their visual prognosis remains unknown. Dry age-related macular degeneration, hypertensive retinopathy, and myopic fundus were associated findings in eight, four, and one patient(s), respectively.

The most frequent stages of SICS during which rent occurred included nucleus prolapse and delivery (seven times), capsulorrhexis (six times), and cortex wash (six times). Rent also occurred during hydro dissection in one case (Table 1).

**Table 1:** Stage of surgery in which rent occurred for SICS cases SICS: small incision cataract surgery; IOL: intraocular lens

Stage of surgery	Frequency	Percentage
Capsulorrhexis	6	20.69
Hydrodissection	1	3.45
Nucleus prolapse and delivery	7	24.13
Cortex wash	6	20.69
IOL implantation	0	-
Visco wash	0	-

The stages of phacoemulsification at which PCR occurred included nucleus management followed by injection of visco (three and two cases). Additionally, PCR was also seen during capsulorrhexis, hydro dissection, irrigation and aspiration, and during the removal of ophthalmic viscosurgical devices (OVDs), with one case each (Table 2).

**Table 2:** Stage of surgery in which rent occurred for phacoemulsification cases OVD: ophthalmic viscosurgical device; CTR: capsular tension ring

Stage of surgery	Frequency	Percentage
Capsulorrhexis	1	3.45
Hydrodissection	1	3.45
Phacoemulsification	3	10.34
Irrigation and aspiration	1	3.45
Injection of visco	2	6.9
Removal of OVD	1	3.45
CTR fixation	0	-

Most cases (15 cases) with PCR were managed by performing anterior vitrectomy and putting the IOL in the sulcus, followed by anterior vitrectomy and the IOL in the bag (six cases), the IOL in the sulcus without vitrectomy (one patient), and posterior vitrectomy in one case. Anterior vitrectomy with an iris-claw was performed in four patients. Two patients were left aphakic and needed a second surgical intervention; one of them underwent pars plana vitrectomy with scleral-fixated IOL on post-op day (POD) 30, and the other had an iris-claw placed on POD 3 (Table 3).

**Table 3:** Management of PCR PCR: posterior capsular rent; IOL: intraocular lens; V: vitrectomy

Vitrectomy+IOL position	Frequency	Percentage
V+IOL in bag	6	20.69
V+IOL in sulcus	15	51.72
IOL in sulcus	1	3.45
Post V+IOL in sulcus	1	3.45
V+iris claw	4	13.8
Aphakia	2	6.9

All the patients received prednisolone acetate 1% + gatifloxacin 0.30% topical eye drops six times a day in tapering doses, along with nepafenac 0.30% three times a day for one month in the operated eye. Patients achieved a post-op BCVA of 6/60-6/24, 6/18-6/9, and 6/9-6/6 in six, two, and one patient on POD 1; 6/18-6/9 in 15, 17, and eight on POD 7; and 6/9-6/6 in eight, 10, and 20 patients on POD 30, respectively (Table 4).

**Table 4:** Visual acuity assessment The above table shows the number of patients falling under each category preoperatively, on postoperative day (POD) 1, POD 7, and POD 30, respectively. BCVA: best corrected visual acuity; PL + PR: perception of light + projection; HMCF: hand movements close to face; CF1M: counting fingers at 1 meter

BCVA	Preop	POD1	POD7	POD30
PL+ PR accurate	4	0	0	0
HMCF-CF1M	10	0	0	0
6/60-6/24	5	6	2	1
6/18-6/9	9	15	17	8
6/9-6/6	1	8	10	20

Data analysis revealed that the Z value was ‑4.418, and the P value was less than 0.001, which was statistically significant (Table 5). None of the patients had any postoperative complications.

**Table 5:** Visual acuity on preoperatively and on day 30 Z value was -4.418 and P value was <0.001. Wilcoxon signed-rank test was used and a statistically significant difference in patient visual acuity preoperatively and on postoperative day 30 was observed. PL + PR: perception of light + projection; HMCF: hand movements close to face; CF1M: counting fingers at 1 meter

	Preoperative		Postoperative day 30	
	Frequency	Percentage	Frequency	Percentage
PL+PR accurate	4	13.8	0	0
HMCF-CF1M	10	34.5	0	0
6/60-6/24	5	17.2	1	3.4
6/18-6/9	9	31	8	27.6
6/9-6/6	1	3.4	20	69
	29	100	29	100

## Discussion

The average age of the patient was found to be 67.76 years, which was close to that found in studies by Amin et al., Pradeep et al., and Segers et al., in which the average ages were 66.62, 65.5, and 74.8±10.5 years, respectively [8-10]. In our study, 16 out of 29 patients were male, which is 55.2%, similar to the findings of studies done by Pradeep et al. and Amin et al., who found the incidence of PCR to be higher in males [9,11]. However, this was in contrast to studies conducted by Amin et al. and Segers et al., who found more prevalence in females, 53% and 55.5%, respectively [8,10]. Various studies were done for risk factor analysis, both systemic and local; this includes a study done by Keles et al. examining pseudo exfoliation, one of the other risk factors, which was found in 3.45% of our cases [12]. A study done by Segers et al. showed corneal opacity and white cataracts to be associated, which was consistent with our finding [10].

Studies by Amin et al. and Keles et al. showed that PCR occurs more often in extracapsular cataract extraction than in phacoemulsification [11,12]. This supports our result, because in our study, among the total PCR cases, 68.97% were SICS and 31.03% were phacoemulsification cases (although PCR is believed to occur more commonly in phacoemulsification than in SICS, phacoemulsification, as a procedure being an independent risk factor is not yet proven). Out of the total number of cases, PCR occurred slightly more often in the patients of inexperienced surgeons (51.7%) than of experienced ones (48.3%), similar to the study done by Pradeep et al. where he observed that the percentage, with respect to the total number of cataracts, was higher in resident-operated cases compared to faculty-operated cases: 1.96 and 1.46, respectively [9]. Chakrabarti and Nazm and Amin et al. also observed a greater number of cases with novice surgeons [5,11].

Research is available on the relation between posterior polar cataracts and posterior capsule deficiency, including the studies conducted by Pujari et al. and Kymionis et al. [13,14]. However, in our study, no true case of posterior polar cataract was encountered. The most prevalent cataract was the posterior subcapsular type (65.5%), followed by immature senile, hyper mature cataract, and pseudo posterior polar cataract, respectively. In our study, we observed the highest number of cases during nucleus prolapse and delivery in SICS and nucleus management in phacoemulsification. This was parallel with the observations of Osher et al. [15].

A similar record-based study conducted by Al-Saffar and Hama found that the incidence of PCR was 5.4%, of which 55.17% underwent primary IOL implantation, 27.5% had secondary IOL implantation, and 17.24% were left aphakic [1]. In this study, the percentage of patients who were left aphakic and hence in need of secondary intervention was found to be 6.9%, and the other 93.1% of cases were managed on the same day and same occasion. In this study, 20.7% of the patients had in-bag implantation of the IOL. The study showed most of the patients having BCVA 6/18-6/9 in POD 1 (58.6%) and POD 7 (58.6%), and most patients having BCVA of 6/9-6/6 by POD 30 (68.97%), which was similar to a study after two months of follow-up by Amin et al. who saw normal BCVA in 78% of his patients [8]. However, a study done by Pradeep et al. revealed BCVA of more than 6/18, only in 19% of cases on POD one [9].

The limitation of the study had a small sample size, and the duration of follow-up was relatively short.

## Conclusions

In our study, PCR was more common in males, with an average age of 67.76 years. However, no clear correlation could be made, because the total demographic distribution of the institution is not known. Incidence was higher in patients with no known ocular surgery or procedure in the past, which might have led to more anxious and uncooperative behavior during surgery. SICS resulted in more PCR cases than phacoemulsification, with a similar number seen in both experienced and inexperienced surgeons. However, confounders such as the allotment of complicated cases need to be determined. Most of the patient’s anterior segments were normal. However, pterygium and corneal opacity were seen, which can cause poor visibility; small pupil and shallow anterior chamber, which can reduce working space; and myopia and pseudoexfoliation. The type of cataract was also studied; however, no true posterior polar cataract was seen in this study.

PCR appeared to occur more frequently when nucleus delivery, capsulorrhexis, and cortex wash were performed during SICS, and in the step of nucleus management in phacoemulsification. Most of the patients underwent anterior vitrectomy, and the IOL was in the sulcus in most cases, followed by the bag itself and the iris claw in a few cases. Only two patients were left aphakic, and a secondary procedure was needed. However, the postoperative visual outcome was satisfactory in most cases, including patients with vitreous loss, most showing 6/9-6/6, whereas those with lower BCVA were associated with posterior segment pathology. Thus, PCR is preventable with meticulous planning and can be effectively managed with early detection during the procedure.
